# Evaluating the influence of phosphorus supply on photosynthetic and agronomic performance of two breeding lines of common bean grown under acidic soil and high temperature stress

**DOI:** 10.1371/journal.pone.0324863

**Published:** 2025-05-28

**Authors:** Juan Carlos Suárez, José Alexander Anzola, José Ivan Vanegas, Amara Tatiana Contreras, Idupulapati M. Rao

**Affiliations:** 1 Programa de Ingeniería Agroecológica, Facultad de Ingeniería, Universidad de la Amazonia, Florencia, Colombia; 2 Centro de Investigaciones Amazónicas CIMAZ Macagual César Augusto Estrada González, Grupo de Investigaciones Agroecosistemas y Conservación en Bosques Amazónicos-GAIA, Florencia, Colombia; 3 International Center for Tropical Agriculture (CIAT), Cali, Colombia; University of Texas at Brownsville: The University of Texas Rio Grande Valley, UNITED STATES OF AMERICA

## Abstract

High temperature stress has a significant effect on the physiological response of the bean crop (*Phaseolus vulgaris* L.) and the supply of phosphorus (P) can influence photosynthetic performance, mobilization of photoassimilates and alleviate heat stress. The objective of this study was to evaluate the influence of increasing P supply on the response of the photosynthetic apparatus of two breeding lines of common bean grown in acidic soil under high temperature stress conditions in a screenhouse. A completely randomized block design with factorial arrangement was used: i. five levels of P supply (P0, P15, P30 and P45 kg ha^-1^; and P supplied through organic matter [PSOM at P25]), and ii. two bean lines (BFS 10, SEF 10) with a total of ten treatments and four replications. During the study, the ambient temperature rose to 37°C and 29°C during the day and night, respectively, a high temperature stress condition that significantly affected the functioning of the photosynthetic apparatus of the two bean lines evaluated. Under these growing conditions, the bean lines adjusted in canopy temperature, reducing leaf temperature by 3.6 to 4.0°C compared to ambient temperature, a process performed more efficiently by SEF 10 compared to BFS 10. Increased P supply improved electron transport chain function, ATP production, PSII photochemical efficiency (F_v_/F_m_), the fraction of energy devoted to the photosynthesis process (ΦII); and reduced the amount of energy in the form of heat (ΦNPQ) as the need for heat dissipation manifested through leaf temperature difference (LTD). These adjustments to photosynthetic apparatus translated into superior agronomic performance through greater partitioning of dry matter into grain yield (GY) as revealed by partitioning indices such as pod partitioning index (PPI), pod harvest index (PHI) and harvest index (HI), and yield components including pod number per area (PNA) and seed number per area (SNA). Increased P supply increased leaf P concentration and alleviated the effects of high temperature on the functioning of the photosynthetic apparatus in both common bean lines (BFS 10 and SEF 10); and accumulation of sugars in pods and seeds facilitated improved seed yield. These two bean lines can serve as parents in bean breeding programs that aim to combine low P tolerance with high temperature tolerance in acid soil regions of the tropics.

## 1. Introduction

Common bean (*Phaseolus vulgaris* L.) is one of the most important food legumes for direct human consumption [1,2]. This crop is mostly produced by smallholder farmers in developing countries in Latin America and Africa accounting for about 75% of global production [3]. However, about 60% of the bean crop area presents problems of low soil fertility associated mainly with nutrient deficiencies, particularly low availability of phosphorus (P) in soil [4,5]. Low fertility of acidic soils in the tropics is the main cause of the yield gap and low P availability represents the most limiting factor for crop development [6]. In addition to these limitations imposed by low soil fertility, temperature increases of 2°C have been estimated, which can generate heat waves that can make 50% of the common bean growing areas unsuitable for cultivation by 2050 [7–9].

Common bean is a nutrient-demanding crop due to its sensitivity to environmental stress [10]. Specifically, sensitivity to above-optimal temperatures and P deficiency negatively affect photosynthetic processes, growth, and grain yield of bean crop [11]. Photosynthetic functioning is known to be one of the most sensitive physiological processes to high temperatures, and common bean is more sensitive to high temperature than the other grain legumes [12]. Heat stress in bean generates a series of irreversible damages in plant metabolism and development [13–16]. Specifically, CO_2_ fixation and photosystem II (PSII) complexes of the photosynthetic apparatus are more sensitive components to high temperatures [17]. Occurrence of high temperature extremes could negatively impact virtually all aspects of photosynthetic activities, such as i) changes in enzymatic activities and biochemical reactions, ii) damage to the ultrastructure of the chloroplast, thylakoid membrane, and PSII centers, iii) damage to PSII proteins, iv) alterations in membrane fluidity and photosystem (PS) protein complexes, and v) up to irreversible destruction of the entire PSII complex [18]. These changes often result in a decrease and/or loss of productivity due to reduced carbon assimilation and biomass accumulation impacting on grain yield [15,19].

In the case of P, as an essential nutrient, it has a central role in energy conservation and transfer in cellular metabolism [20], as well as growth and yield in grain legumes such as beans [5]. At the physiological level, P plays key role in production of several compounds such as ATP, NADPH, nucleic acids, sugar phosphates, and phospholipids, which play important roles in photosynthesis [20]. However, the availability of P in tropical soils does not meet the demand required by beans [21], and the photosynthetic performance is sensitive to low P levels [22]. Photosynthetic capacity in low P leaves is affected through i) low ATP production, ii) decrease in RuBP regeneration, and iii) decrease in RubisCO activity that catalyzes CO_2_ fixation [23,24]. This leads to reduced photoassimilates that limit leaf expansion and growth, thus presenting yield reductions [25]. Thus, both heat and low P stress situations strongly affect chlorophyll fluorescence processes in PSII reaction centers, chloroplast and cell membrane integrity [26], in the short and relatively long-term, thereby causing a reduction in quantum yield of PSII (F_v_/F_m_), electron transfer rate (ETR), photochemical quenching coefficient (qP), and PSII effective quantum yield (ΦII) [27]. In this sense, combined stress of heat and low P in common bean, can probably be mitigated by developing photosynthetically adapted genotypes [28]. Thus, it is necessary to understand the mechanisms and/or physiological strategies of plants, and this knowledge is necessary to develop breeding strategies for improved adaptation to combined stress conditions [5,13,29].

Heat stress can alter photoassimilates partitioning, favoring the accumulation of sugars in leaves rather than their translocation to reproductive organs, which negatively affects seed filling and final crop yield [30]. However, adequate P supply can mitigate these negative effects by stabilizing cell membranes and regulating heat stress-sensitive metabolic processes such as photosynthesis and respiration [31]. Under conditions of high temperature stress, an optimal P supply improves photosynthetic efficiency by maintaining chloroplast integrity and increasing the synthesis of phosphorylated compounds needed for energy production [24,32]. This allows plants to maintain higher levels of carbohydrates even under heat stress conditions. It has been observed that accumulation of carbohydrates under heat stress conditions is an adaptive trait associated with heat stress tolerance [33].

Previous studies have found that under high temperature conditions, plants tend to increase P uptake under adequate P supply leading to higher P concentration in leaf tissue [34]. However, there is limited knowledge on how increasing P supply interacts with temperature for alleviating the negative effect of high temperatures on photosynthetic performance [11]. In this sense, in the Colombian Amazon region, studies have been conducted for identifying genotypes that adapt to acid soils with low fertility and high temperature stress [15,16,35]. Some bean lines have been identified for their outstanding performance in terms of grain yields under these conditions, most notably BFS 10 (*P. vulgaris*) and SEF 10 (cross of *P. vulgaris* × *P. acutifolius* × *P. coccineus*) of Mesoamerican bean type that presents a higher concentration of iron (Fe) and zinc (Zn) in seeds and that also agronomically perform as well compared to standard varieties, due to a combination of desirable characteristics that include good yield, grain color, grain size and shape, characteristics desired by farmers [16,36,37].

In our previous studies [16,35], we used different physiological variables related to the functioning of the photosynthetic apparatus, which mainly focus on energy use in leaves (i.e., energy passed to photochemistry (ΦII), dissipated as heat (ΦNPQ) and dissipated in an unregulated manner (ΦNO)), and leaf cooling as in the functioning of reaction centers. Using this approach, it has been possible to observe physiological adjustments in two common bean lines to alleviate high temperature stress conditions with an increase in P supply [38]. This study was aimed to (1) evaluate the response of the photosynthetic apparatus to an increase in P supply as well as its influence on soluble sugar accumulation and mobilization to seeds; (2) test the relationships between photosynthetic characteristics and agronomic performance of two breeding lines of common bean grown on acid soils under high temperature stress conditions. High temperature stress conditions were induced under screenhouse conditions to simulate the field conditions of the smallholder bean growers in the Amazon region. We tested the hypothesis that sufficient supply of P improves photosynthetic and agronomic performance of acid soil adapted common bean genotypes grown under high temperature stress conditions.

## 2. Materials and methods

### 2.1 Study site and experimental design

**Fig 1:**
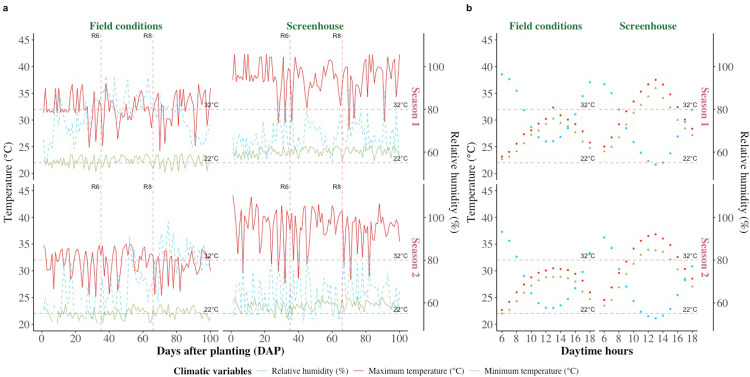
Changes in maximum and minimum temperature, and relative humidity during the two seasons (2019) and (2020) under outside (field conditions) and inside (screenhouse) conditions at the Macagual Research Center in Colombia. a. Changes during the experimental period in the two seasons. b. Changes during daytime in two seasons. The dotted horizontal line denotes 22°C and 32°C that are considered critical limits for night and day temperatures for growth and productivity of common bean.

The study was conducted in a screenhouse located at the Macagual Amazon Research Center of the University of the Amazon (1°37’ N 75°36’ W), with the objective of inducing combined stress conditions of high temperature and acidic soil. The research covered two periods: from October 2019 to January 2020 and from September to December 2020, with an average ambient temperature inside the screenhouse of 28.3 ± 0.1°C and 28.1 ± 0.1°C, respectively, which was on average 2.9°C higher than what was observed outside of the screenhouse under field conditions. The relative humidity (RH) inside the screenhouse for the same periods was 77 ± 0.3% and 75 ± 0.3%, respectively, which was 9% lower than under field conditions. The daytime temperature inside the screenhouse ranged from 24.1°C to 37.9°C, and the nighttime temperature ranged from 23.9°C to 27.3°C, with an average relative humidity of 67% during the day and 87% at night (Fig 1). Weather conditions were recorded at each site using WatchDog 2900ET weather stations (Spectrum Technologies, Inc., USA).

For this study, a completely randomized block design was implemented under a factorial arrangement where two factors were considered: i) five levels of P supply and ii) two bean lines, for a total of 10 treatments with four replications. The first factor was the supply of P (kg ha^−1^) through the application of rock phosphate (RP) in four different amounts (P0, P15, P30, P45), as well as the application of P contained in soil organic matter (PSOM) corresponding to 25 kg P ha^−1^ (P25), treatments that were compared to the control with no application of P (P0), which corresponded to plants that did not receive any P supply. The second factor consisted of two interspecific bean lines of Mesoamerican germplasm with indeterminate growth habit type II, BFS 10 [(SER 76 × RCB 589)_F1_ × (SXB 407 × SER 119)] and SEF 10 [(ALB 74 × INB 841)_F1_ × RCB 593] [38]. Seeds of the two common bean lines used in the study were provided by the Alliance of Bioversity International and International Center for Tropical Agriculture (CIAT) Bean Breeding Program. Each block (n = 4) was a concrete bed of 1.5 m wide by 8 m long with a depth of 0.4 m at a height of 1.2 m, which contained an acidic Oxisol of clay loam texture (pH = 4.2, aluminum saturation = 73.4%, exchangeable Al = 6.3 cmol(+) kg^−1^, exchangeable acidity = 1.76 cmol kg^−1^) with low soil fertility (organic carbon = 3.6%, available P (Bray-II) = 1.04 mg kg^−1^, cation exchange capacity = 17.5 cmol kg^−1^), and low total base saturation of 3.9% (cmol kg^−1^: Ca: 0.25, Na: 0.24, K: 0.09, Mg: 0.11). Four rows of 8 m in length were planted, spaced at 0.4 m between rows, and within each row, plants were spaced at 0.1 m apart, equivalent to 15 plants m^−2^, where each treatment consisted of 32 plants. For more details on the chemical composition and the quantities applied from the P sources, as well as the experimental management, see Suárez et al. [38].

The phosphorus (P) supply treatments for this study were designed following published reports on tropical soils cultivated with leguminous crops, such as beans [39,40]. These P treatments are selected to provide four levels of P availability: deficient, low, moderate and high, corresponding to application rates of 0, 15, 30 and 45 kg ha  ¹ of P, respectively. To provide these different levels of P supply, rock phosphate (RP) “Phosphorite, INFERHUILA S.A.” was used as the inorganic P source. This material is particularly suitable for legume production in acidic soils due to its composition, which includes 3% readily assimilable P (P₂O), 21% slow-assimilation P (P₂O), and 32% calcium (CaO). The RP was applied at total rates of 500, 1000, and 1500 kg ha  ¹, corresponding to P supply levels of 15, 30, and 45 kg ha  ¹, respectively. For the organic P treatment, P was supplied through organic matter [PSOM at P25]. No additional fertilizers were applied, and the plants were carefully protected from pests and diseases throughout the experiment. To ensure that water availability did not limit reproductive development, the plants were irrigated daily using a drip irrigation system at field capacity. Soil moisture content was continuously monitored with an EC-5 soil moisture sensor (Decagon Devices, Inc).

### 2.2 Distribution of energy use, photosynthetic apparatus functioning, and leaf cooling capacity under high temperature stress conditions

The monitoring of the functioning of the photosynthetic apparatus was carried out during two seasons (2019, 2020), as well as in two phenological stages (i. flowering: BBCH 60, ii. mid-pod filling: BBCH 79, at 35 and 65 days after planting, respectively) during each crop growing season. In each plot, four plants were taken from the central row, and from each plant, a leaf was taken from each stratum (lower, middle, upper) of the canopy. This process was conducted at different times of the day (06:00, 08:00, 12:00, 14:00, 16:00, 18:00 solar time). The MultispeQ V2.0 (PhotosynQ INC, East Lansing, Michigan, USA) was used to evaluate differences in the functional status of the photosynthetic apparatus. This device measures various physiological and environmental variables, enabling the determination of the influence of varying P supply on the functional status of the photosynthetic apparatus under high-temperature stress conditions. Each measurement was performed using an Android mobile device connected to the MultispeQ instrument via Bluetooth. A total of 1680 measurements were collected and stored in the PhotosynQ platform [41] without presenting error-logged data during sampling.

We focused on evaluating 28 variables using the MultispeQ V2.0, which include environmental, thermal (at leaf level), morphological, fluorescence, electron transport, and absorbed light direction variables, as well as the functioning of ATP synthase and PSI. Major environmental variables measured include relative humidity (RH), ambient temperature (T_a_), and photosynthetically active radiation (PAR). Regarding thermal variables at the leaf level, leaf temperature (LT) and leaf temperature difference (LTD, calculated as the difference between LT and ambient temperature, T_a_) were measured. Among the morphological variables, leaf thickness ((Thi) in micrometers [42]) was determined. The relative chlorophyll (RC) as well as the different variables related to chlorophyll *a* fluorescence (Chl_a_) were determined as follows: F_o_ (minimum fluorescence), F_m_ (maximum fluorescence), F_s_ (steady-state fluorescence), and F_v_/F_m_ (PSII efficiency) were measured, providing necessary information to calculate electron transport and the direction of absorbed light. These are related to quantum yield of PSII electron transport (ΦII), the portion of energy wasted through unregulated photosynthetic processes that pose a potential risk to the plant (ΦNO), and the fraction of light dedicated to non-photochemical quenching, which dissipates energy as heat (ΦNPQ) [43]. Additionally, total non-photochemical quenching of absorbed light energy (NPQ_t_) [44], the fraction of open PSII reaction centers (qL) [42], and linear electron flow (LEF) [45] were also measured.

The functioning of ATP synthase (vH+) was determined by measuring the rate of proton flow through the thylakoid membrane (i.e., proton motive force [*pmf*]), which is an important variable in the dark phase of photosynthesis, specifically in the Calvin cycle, as it depends on the amount of ATP produced to form photoassimilates [46]. Additionally, the proton conductivity of ATP synthases in the thylakoid membrane (gH + ; [47]) and the maximum amplitude of electrochromic shift (ECS_t_), which reflects changes in the electric field across the thylakoid membrane, or light-induced pmf, were calculated. The state of the photosystem I (PSI) reaction centers was also measured, including active PSI centers (PSI_act_), the fraction of oxidized PSI centers (PSI_ox_), and PSI in the open state (PSI_open_), as well as over-reduced PSI (PSI_or_) [48]. Moreover, the electron transfer rate constant in P700 (_k_P700), the initial electron transfer rate in steady-state P700 (_i_P700), the electron transfer lifetime of P700 (_t_P700), and the amplitude of P700 electron transfer during the light-dark transition (P700_Da_), which indicates the capacity of PSI to transfer electrons, were also calculated [48].

### 2.3 Determination of soluble sugar levels in plant parts and total phosphorous in leaves

Total soluble sugar concentration (mg g^-1^) in different plant organs (root sugar concentration, RSC; stem sugar concentration, SSC; leaf sugar concentration, LSC; pod sugar concentration, PSC) was determined in the samples from two bean lines (BFS 10 and SEF 10) both at flowering and mid-pod filling growth stages using the phenol-sulfuric colorimetric method [49]. For this purpose, approximately 10 mg of fresh weight of each plant organ was used after homogenization in liquid nitrogen and incubated in a water bath at 75°C for 15 min with 1 mL of 80% (v/v) ethanol. The extract was centrifuged at 12,000 rpm for 15 min, and the supernatant was collected, and the sediment was re-extracted with 500 µL of 80% (v/v) ethanol and combined with the supernatant of the first extraction. For quantification, 250 µL of the samples were mixed with 250 µL of 80% ethanol (v/v), 2.5 mL of concentrated sulfuric acid and 0.5 mL of 5% phenol (v/v). After mixing, the solutions were allowed to stand at room temperature for 20 min, and absorbance was measured at 490 nm using spectrophotometry. A standard curve was established using 1 mg ml^-1^ D-glucose, and the concentration of soluble sugars was expressed as milligrams of per gram fresh weight (mg g^-1^ FW).

The total P concentration in leaves (leaf phosphorus concentration, LPC, mg g  ¹) was determined in two bean lines (BFS 10 and SEF 10) during the mid-pod filling stage using the colorimetric technique of Murphy and Riley [50]. A 0.5 g dry sample was weighed and subjected to acid digestion using 5 mL of nitric acid (HNO_3_) and 2 mL of perchloric acid (HClO₄) on a heating plate until a clear solution was obtained, which was then diluted to 50 mL with distilled water. For quantification, phosphate standard solutions (0–25 µg P mL^-1^) were prepared, and the characteristic blue color was developed by adding 5 mL of ammonium molybdate reagent and 1 mL of ascorbic acid, allowing the mixture to rest for 30 minutes before measuring the absorbance at 660 nm in a spectrophotometer. Similarly, a 5 mL aliquot of the digested extract from the samples was treated with the same reagents. The total P concentration was calculated using the calibration curve and expressed as milligrams of P per gram of dry weight (mg g^-1^ DW)

### 2.4 Data analysis

Each of the environmental, thermal (at leaf level), morphological, fluorescence, electron transport and absorbed light direction variables, as well as the functioning of ATP synthase and PSI (S1 Table) were analyzed using linear mixed models (LMM). The LMM had fixed factors of the five levels of P supply (P0, P15, P30 and P45 kg ha^-1^; and P supplied through organic matter (PSOM)) and the two bean lines (BFS 10 and SEF 10). The random factors included in the model were the block within the plant stratum, time of the day, growth stage and the evaluation period in each season. The Akaike information criterion (AIC) and the Bayesian information criterion (BIC) were used to select the model for each of the variables, considering the assumptions of normality and homogeneity of variance. Statistical differences in means were evaluated using the LSD Fisher test using an error level of 5%. With the information obtained, box plots were drawn up and a correlation analysis was made between the different physiological variables analyzed in this study and the different agronomic and biomass partitioning variables determined from the experimental study conducted by Suárez et al. [38]. Likewise, a comparison analysis was carried out in relation to the percentage change by each factor of the different parameters of photosynthetic apparatus functioning of two common bean lines grown under high temperature stress conditions. For this purpose, the percentage difference between the level of P supply of P45 in relation to P0 and the percentage difference between SEF 10 in relation to BFS 10 were considered. The lme function of the nlme package was used to perform the LMMs, and the graphical outputs were performed in the packages “ade4”, “ggplot2”, “factoextra” and “corrplot” in the R language software, version 4.4.2 [51].

## 3. Results

### 3.1 Influence of P supply on leaf cooling ability of two bean lines to cope with high temperature stress

**Fig 2:**
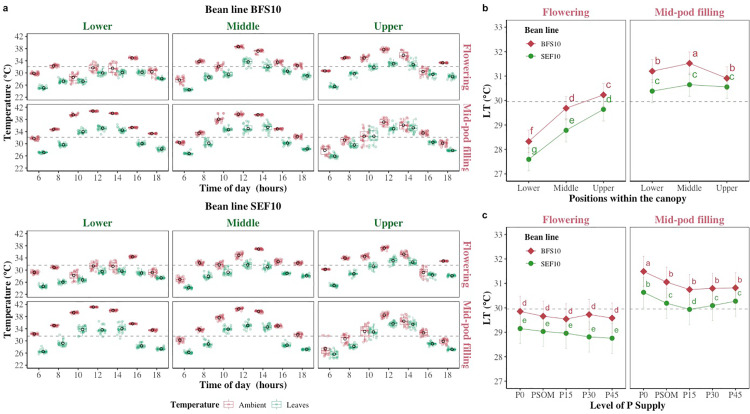
Ambient temperature and leaf temperature differences observed with two bean lines (BFS 10, SEF 10). a. Ambient and leaf temperature (LT°C) changes in three different strata (Lower, Middle, Upper) of the canopy and at two growth stages (Flowering, Mid-pod filling) in two bean lines (BFS 10, SEF 10). Values are mean and standard error in each hour (n = 40, four replications under five levels of P supply in two seasons). b. Leaf temperature (LT°C) differences in three different strata (Lower, Middle, Upper) at two different growth stages (Flowering, Mid-pod filling) in two bean lines (BFS 10, SEF 10). Values are mean and standard error of LT (n = 320, four replications in eight monitoring periods during the day under five levels of P supply in two seasons), c. Leaf temperature (LT°C) differences at two growth stages (Flowering, mid-pod filling) in two bean lines (BFS 10, SEF 10) with different P supplies (P0, P15, P30 and P45 kg ha^−1^; and P supplied through organic matter (PSOM)) to acidic soil under high temperature stress conditions. Values are mean and standard error (n = 192, four replicates in eight monitoring periods during the day in three strata of the canopy in two seasons). The dashed black line in each panel denotes the mean of each variable. ^a, b, c^: Means with a letter in common between factors are not significantly different based on the LSD means test (p < 0.05).

The ambient temperature measured during sampling inside the screenhouse reached maximum values of 39.1°C and 41.2°C during the flowering and the mid-pod filling growth stages, respectively, with average values of 29.1 ± 0.1°C and 29.9 ± 0.2°C to 35.2 ± 0.2°C and 39.6 ± 0.1°C at 06:00 and 12:00 hours, respectively. Environmental conditions significantly influenced leaf temperature, and this was dependent on time of the day, stratum (upper, middle, lower) of the canopy (Fig 2a), growth stage in phenological development of each bean line (Fig 2b), and the level of P supply (Fig 2c) (P < 0.05). It was found that BFS 10 and SEF 10 had a leaf temperature difference (LTD) of 3.61 ± 0.06°C and 3.98 ± 0.07°C between leaf temperature relative to ambient temperature regardless of stratum of the canopy, time, phenological stage, and level of P supply. At the stratum level the average LTD value found at the lower, middle and upper part of the canopy was 4.42 ± 0.09°C, 4.14 ± 0.06°C and 2.83 ± 0.08°C, respectively, being able to dissipate more the effect of ambient temperature by SEF 10 compared to BFS 10 by 0.56°C at the lower, 0.4°C in the middle and 0.16°C in the upper (Fig 2a) level of the plant canopy. A greater temperature difference was found at the mid-pod filling growth stage (4.03 ± 0.07°C) compared to that of flowering (3.56 ± 0.07°C) regardless of the other factors. When comparing the differences between the two bean lines, on average BFS 10 at both flowering (29.4 ± 0.1°C) and mid-pod filling (31.1 ± 0.1°C) growth stages was 0.72°C and 0.68°C above SEF 10, respectively, in all three strata of the canopy (Fig 2b, P < 0.05). On the other hand, a significant effect on LTD was found by increasing P supply, being very evident the difference between the two bean genotypes in different phenological stages (Fig 2c, P < 0.05).

**Fig 3:**
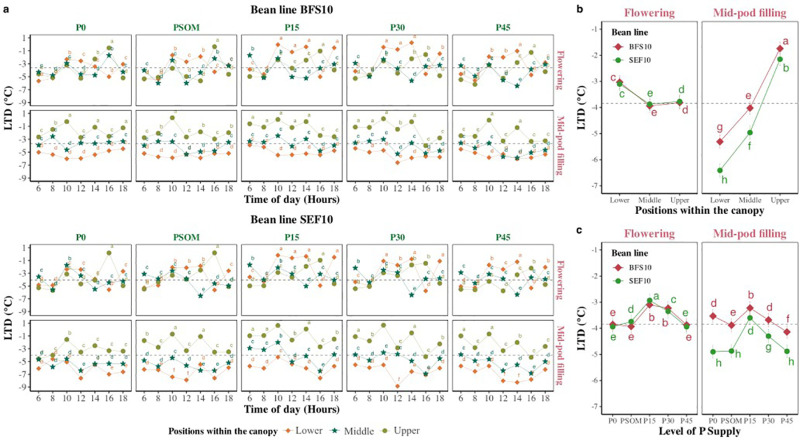
Changes in leaf temperature difference (LTD°C) in two bean lines (BFS 10, SEF 10) as influenced by increasing P supply. a. Changes in LTD under five levels of P supply in two bean lines. Values are mean and standard error in each hour (n = 24, four replications in three canopy strata (Lower, Middle, Upper) in two seasons). b. Leaf temperature difference (LTD°C) changes in three different strata (Lower, Middle, Upper) at two different growth stages (Flowering, Mid-pod filling) in two bean lines (BFS 10, SEF 10). Values are mean and standard error of LTD (n = 320, four replications in eight monitoring periods during the day under five levels of P supply in two seasons), c. Leaf temperature difference (LTD°C) changes at two growth stages (flowering, mid-pod filling) in two bean lines (BFS 10, SEF 10) with different P supplies (P0, P15, P30 and P45 kg ha^−1^; and P supplied through organic matter (PSOM)) to acidic soil under high temperature stress conditions. Values are mean and standard error of leaf temperature difference (LTD°C) (n = 192, four replicates in eight monitoring periods during the day in three strata of the canopy in two seasons) at two different plant growth stages in two bean lines with different P supplies (P0, P15, P30 and P45 kg ha^−1^; and P supplied through organic matter (PSOM)) to acid soil under high temperature stress conditions. The dashed black line in each panel denotes the mean of each variable. ^a, b, c^: Means with a letter in common between factors are not significantly different based on the LSD means test (p < 0.05).

When analyzing the dissipation capacity of the increase in ambient temperature over the canopy, differences were found at the level of bean line, P supply level as well as in the different strata of the canopy at each growth stage of crop development during daily monitoring (Fig 3). For example, in the mid-pod filling growth stage, at the P0 supply level, the lowest value of LTD was presented in SEF 10 with −4.93 ± 0.1°C, however for this same bean line at flowering growth stage at P15 the highest value of LTD was presented with −2.88 ± 0.2°C (Fig 3a). When considering only the stratum of the canopy within the growth phase it was found that the bean lines were different at mid-pod filling, being SEF 10 more efficient compared to BFS 10 to dissipate the ambient temperature (Fig 3b, P < 0.05). On the other hand, LTD value was not found to have a trend in relation to increasing P supply. But variation between the two bean lines was more evident at mid-pod filling growth stage (Fig 3c).

### 3.2 Influence of P supply on photosynthetic performance of two common bean lines under high temperature stress conditions

**Fig 4:**
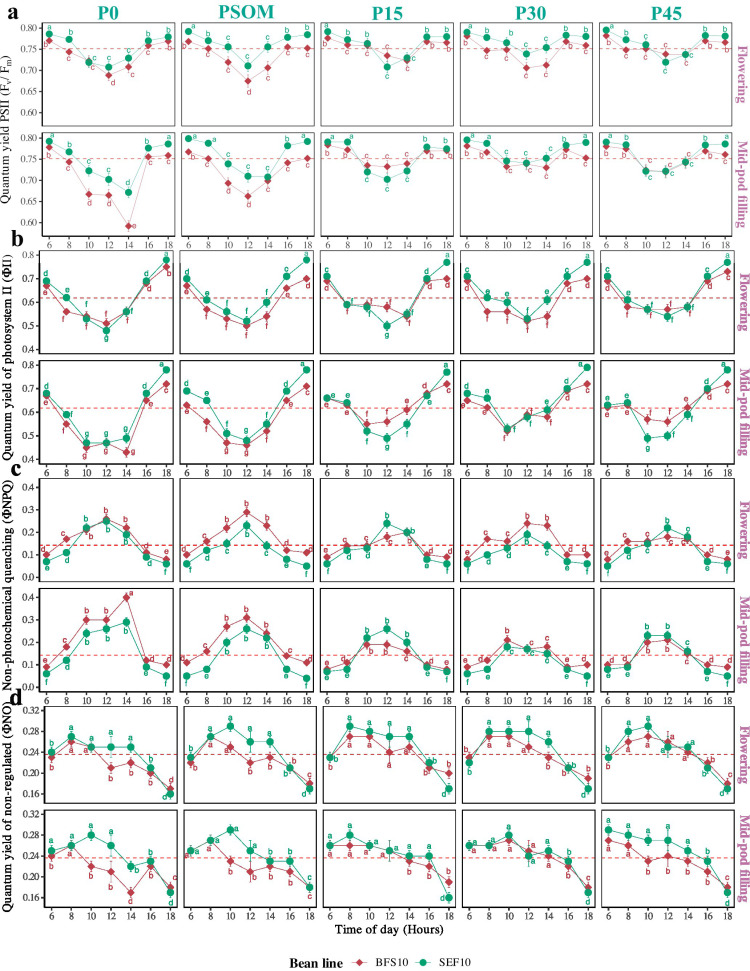
Photosynthetic apparatus functioning and energy fraction distribution at two growth stages (Flowering, Mid-pod filling) of two bean lines (BFS 10, SEF 10) during the daytime as a function of different levels of P supply (P0, P15, P30 and P45 kg ha^-1^; and P supplied through organic matter (PSOM)) to acidic soil under high temperature stress conditions. a, b, c: Means with a letter in common between factors are not significantly different based on the LSD means test (p < 0.05). The dashed line in each panel denotes the mean of each variable.

A significant effect of increased P supply, both at flowering and mid-pod filling growth stages in bean lines, was observed on the functioning and distribution of the energy fraction in the photosynthetic apparatus (Fig 4), where all variables showed significant differences in daily behavior across different factors (P supply level, plant growth stage, and bean line). For example, the PSII quantum efficiency (F_v_/F_m_) at 14:00 hours during mid-pod filling at the P0 supply level was 0.59 ± 0.01 and 0.67 ± 0.01 for BFS 10 and SEF 10, respectively, these being the lowest values recorded in the study for each genotype (Fig 4a). However, when the P supply level was increased, for example to P45 at mid-pod filling growth stage, the quantum efficiency increased by 22% and 7% for BFS 10 and SEF 10, respectively, reaching the value of 0.72 for both bean lines (Fig 4a). When comparing the daily behavior of F_v_/F_m_ between the two bean lines, differences were found at P0 and with PSOM (P < 0.05), with SEF 10 showing higher values compared to BFS 10. However, as the P supply level increased (P15, P30, and P45), no differences were observed between the two bean lines (P > 0.05, Fig 4a). Between the two growth stages, the effect of high temperature was more evident during mid-pod filling for P0 compared to flowering at the same P supply level for F_v_/F_m_ (Fig 4a). Regarding the fraction of energy devoted exclusively to the functioning of the photosynthetic apparatus (ΦII), a significant effect of time of day (P < 0.05) was observed, with contrasting values of 0.52 ± 0.01 and 0.75 ± 0.01 at 12:00 and 18:00, respectively (Fig 4b). For example, under P0 at flowering, at 12:00 hours the lowest energy allocation to ΦII was observed for both bean lines, with an increase of 9% and 16.6% at 14:00 hours for BFS 10 and SEF 10, respectively. However, at mid-pod filling under P0, the effect of high temperature was more evident, with ΦII values remaining low from 10:00 hours, reaching 0.45 ± 0.01 and 0.47 ± 0.01 for BFS 10 and SEF 10, respectively (Fig 4b). At higher levels of P supply, a positive relationship was found between the increase in mean ΦII and the increase in P, an effect that was more pronounced during mid-pod filling growth stage. Furthermore, it was observed that BFS 10 responded more quickly in increasing ΦII compared to SEF 10 at P15 and P45 (Fig 4b). The two bean lines exhibited similar behavior at most P supply levels; however, BFS 10 at P45 was 12% more efficient in allocating energy to ΦII compared to SEF 10 at 12:00 hours during mid-pod filling growth stage (Fig 4b).

In response to high-temperature stress, the two bean lines increased energy dissipation as heat (measured as NPQ), a phenomenon that was particularly pronounced at 14:00 hours under the P0 supply level during mid-pod filling growth stage for BFS 10 (Fig 4c). Although no significant differences were observed in the daily NPQ behavior across two different growth stages, a notable decrease of 33% for BFS 10 and 18% for SEF 10 in the fraction dissipated as heat was recorded under P45 supply compared to P0 during mid-pod filling growth stage. Furthermore, the fraction of unregulated energy did not significantly differ between the two bean lines under different P supplies from 8:00–14:00 hours, remaining consistently low. However, between 16:00 and 18:00 hours, this unregulated fraction decreased further (Fig 4d).

### 3.3 Adjustments in the functioning of the photosynthetic apparatus with increase in P supply as an adaptive strategy to heat stress

**Fig 5:**
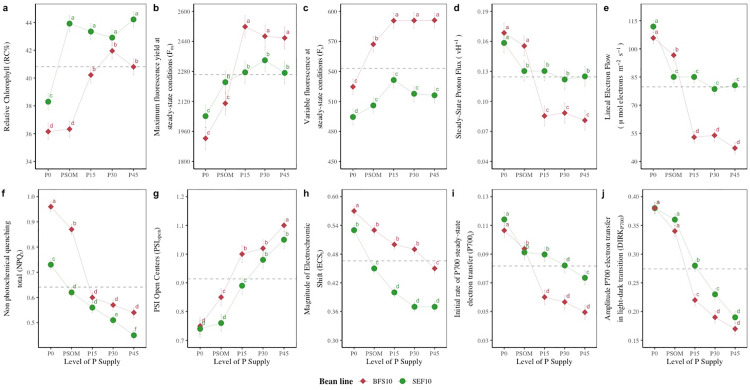
Performance of the photosynthetic apparatus of two bean lines (BFS 10, SEF 10) as a function of different levels of P supply (P0, P15, P30 and P45 kg ha^-1^; and P supplied through organic matter (PSOM)) to acidic soil under high temperature stress conditions. The dashed line in each panel denotes the mean of each variable. ^a, b, c^: Means with a letter in common between factors are not significantly different based on the LSD means test (p < 0.05).

Fig 5 shows the variables that changed with increasing P supply under high temperature conditions. For example, the relative chlorophyll content increased by 12.8% and 15.4% for BFS 10 and SEF 10, respectively, compared to P0. However, in general, the change in chlorophyll concentration was more evident for SEF 10 compared to BFS 10, even with the application of P in the form of organic matter (PSOM, Fig 5a). Regarding maximum fluorescence, there was an interaction at the bean line level as a function of P supply since BFS 10 at both P0 and PSOM presented lower mean values compared to SEF 10. However, when P supply was increased (P15, P30, and P45), BFS 10 showed higher mean values (Fig 5b, P < 0.05). Therefore, it is evident that F_m_ increased by 22.9% when P supply was increased, even under high-temperature stress conditions (Fig 5b, P < 0.05). For variable fluorescence, an effect of increasing P supply was observed, which was more evident in BFS 10 (Fig 5c, P < 0.05). The proton conductance of chloroplast ATP synthase (vH+) was decreased with increasing P supply, with differences between the two bean lines being evident (Fig 5d, P < 0.05). Similar behavior was observed in LEF and NPQ_t_ (Fig 5e and 5f, P < 0.05). The PSI open centers (PSI_open_) increased with P supply, being higher for BFS 10 (Fig 5g, P < 0.05). For the other variables (ECS_t_, P700_i_, DIRK_P700_), these decreased with increasing P supply for both bean lines. However, only for P700i, the SEF 10 bean line showed higher values (Fig 5h–5j, P < 0.05).

### 3.4 Influence of increasing P supply on sugar levels in different plant parts and leaf P concentration under high temperature stress conditions

**Fig 6:**
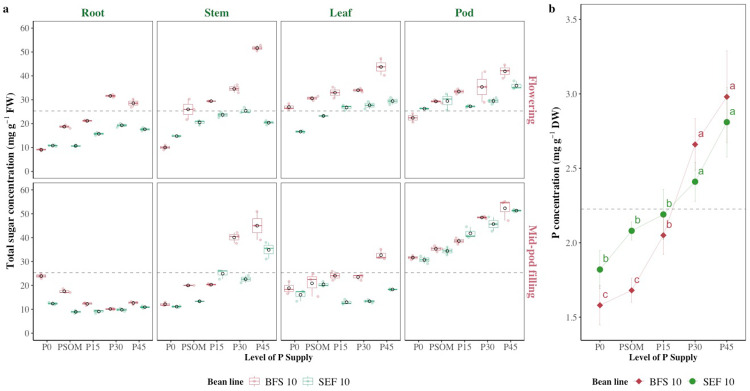
Total sugar and leaf phosphorus concentration of two bean lines (BFS 10, SEF 10) as a function of different levels of P supply (P0, P15, P30 and P45 kg ha^-1^; and P supplied through organic matter (PSOM)) to acidic soil under high temperature stress conditions. a. The total sugar concentration both at flowering and at mid-pod filling growth stages in different plant organs (root, stem, leaf, pod). b. Total phosphorus concentration in the leaf in the mid-pod filling. ^a, b, c^: Means with a letter in common between factors are not significantly different based on the LSD means test (p < 0.05). The dashed line in each panel denotes the mean of each variable.

Total sugar level varied significantly depending on P supply, plant organ, growth stage and bean line (P < 0.05, Fig 6a). In root tissue, values ranged from 8 mg g^-1^ (P0) to 15 mg g^-1^ (P45), with average values of 10−12 mg g^-1^ at P0 and P15, and 13−15 mg g^-1^ at P30 and P45 (Fig 6a). In stem tissue, sugars increased from 10 mg g^-1^ (P0) to 20 mg g^-1^ (P45) at flowering, and up to 30 mg g^-1^at mid-pod filling, with an average range of 12−25 mg g^-1^ (Fig 6a). In leaf tissue, the values were higher at flowering (20−40 mg g^-1^) than at mid-pod filling (15−30 mg g^-1^), with average values of 20−35 mg g^-1^ (Fig 6a). Pod tissue showed higher values, ranging from 20 mg g^-1^ (P0) to 50 mg g^-1^ (P45) at flowering, and reaching 60 mg g^-1^at mid-pod filling, with an average range of 30−55 mg g^-1^ (Fig 6a). Between the two growth stages, mid-pod filling showed higher concentrations, with increases of 50% in the stem tissue and 20% in the pod tissue (P45). Line BFS 10 consistently outperformed SEF 10, with higher values of 20%−33%, particularly in pod (60 mg g^-1^vs. 50 mg g^-1^) and stem (30 mg g^-1^ vs. 25 mg g^-1^) tissues. In general, an increase in P supply (P0 to P45) increased sugars by 50% to 200%, highlighting its impact on reproductive organs and at mid-pod filling growth stage, which underlines the importance of P supply in accumulation of sugars and possible translocation to grain under heat stress (Fig 6a).

Total P concentration in bean leaves varied significantly depending on the level of P supply and the bean line evaluated (P < 0.05, Fig 6b). In general terms, a progressive increase in leaf P concentration was observed in both lines as the level of P supply increased, from P0 to P45. In BFS 10, leaf P concentration increased from 1.5 mg g^-¹^ to 3.5 mg g^-¹^, representing a cumulative increase of 133% (Fig 6b). The SEF 10 showed a more moderate increase, from 1.8 mg g^-1^ to 3.2 mg g^-1^, equivalent to an increase of 77.8%. As for the differences between the two bean lines, under conditions of low P supply (P0 and PSOM), SEF 10 showed a higher P concentration than BFS 10, with differences of 20% and 15%, respectively (Fig 6b). However, at higher levels of supply (P30 and P45), these differences attenuated, reaching similar values in both genotypes, with a minimum difference of 3% within the P45 treatment (Fig 6b).

### 3.5 Relationships between photosynthetic characteristics and agronomic performance with increase in P supply under high temperature stress

**Fig 7:**
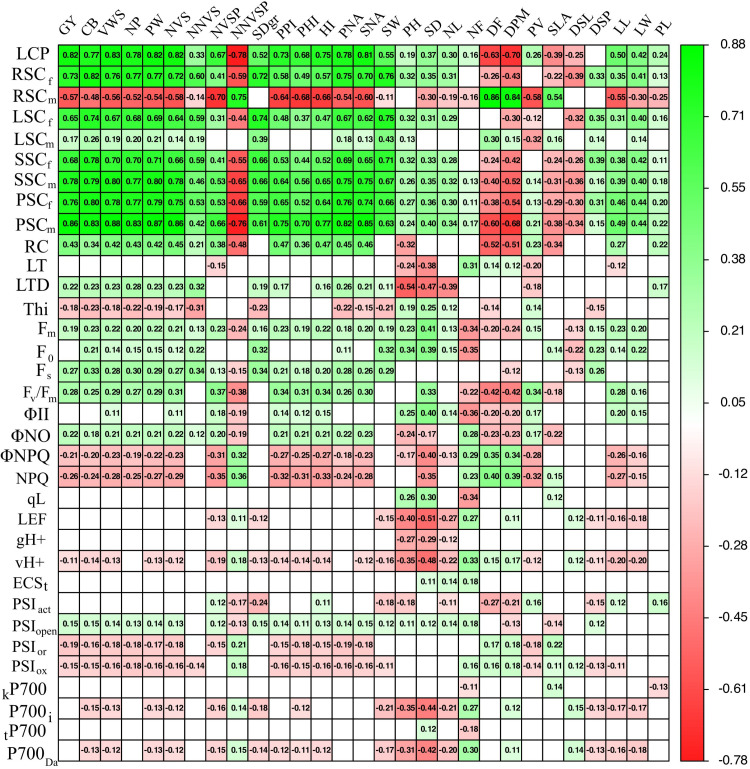
Correlations between different photosynthetic variables with agronomic performance related variables of two bean lines grown in acidic soil under high temperature stress conditions. LPC: leaf phosphorus concentration; RSC: root sugar concentration; SSC: stem sugar concentration; LSC: leaf sugar concentration; PSC: pod sugar concentration; f: flowering; m: mid-pod filling; RC: relative chlorophyll; LT: leaf temperature; LTD: leaf temperature difference; Thi: leaf thickness; F_m_: maximum fluorescence; F_0_: minimum fluorescence; F_s_: steady-state fluorescence; F_v_: variable fluorescence; F_v_/F_m_, maximum quantum efficiency of PSII; ΦII: quantum yield of photosystem II; ΦNO: quantum yield of non-regulated; ΦNPQ: non-photochemical quenching; NPQ_t_: non-photochemical quenching total; qL: proportion of open PSII reaction centers; LEF: linear electron flow; gH + : proton conductivity of ATP synthases in the thylakoid membrane; vH + : the functioning of ATP synthase; ECS_t_: maximum amplitude of electrochromic shift; PSI_act_: total number of active PSI sites; PSI_open_: open state PSI sites; PSI_or_: reduced PSI fraction; PSI_ox_: fraction of oxidized PSI sites. _k_P700: electron transfer rate constant in P700; P700_i_: the initial electron transfer rate in steady-state P700; _t_P700: the electron transfer lifetime of P700; P700_Da_: the amplitude of P700 electron transfer during the light-dark transition. GY: grain yield; CB: canopy biomass; VWS: viable seed weight per plant; NVS: number of viable seeds per plant; NP: number of pods per plant; PW: pod weight per plant; NNVS: number of non-viable seeds per plant; NVSP: number of viable seeds per pod; NNVSP: number of non-viable seeds per pod; PPI: pod partitioning index; PHI: pod harvest index; HI: harvest index; PNA: pod number area; SNA: seed number area; SW: 100-seed weight; PH: plant height; SD: stem diameter; NL: number of leaves; NF: number of flowers; DF: days to flowering; DPM: days to physiological maturity; PV: pollen viability; SLA: specific leaf area; LSD: leaf stomatal density; PSD: pod stomatal density; LL: leaf length; LW: leaf width; PL: pod length; green to red color gradient means positive and negative correlation, respectively. Cells without color mean that there was no statistically significant correlation.

Fig 7 shows the relationship (P < 0.05) between photosynthetic characteristics (rows) with agronomic performance oriented (yield, biomass partitioning and phenological) variables of the two bean lines (columns) with increased supply of P level. The leaf P concentration (LPC, Fig 7) showed positive correlations (P < 0.05) with key agronomic variables related to yield (GY r^2^ = 0.82, PW r^2^ = 0.82, NVS r^2^ = 0.82, VWS r^2^ = 0.83, HI r^2^ = 0.75) and negative correlations with those associated with reproductive limitations, such as NNVSP (r^2^ = −0.76). Additionally, LPC also influenced phenological variables (P < 0.05), accelerating plant development (shorter DF r^2^ = −0.63 and DPM r^2^ = −0.70). High concentration of sugars in roots during flowering (RSC_f_, Fig 7) had a positive impact (P < 0.05) on most agronomic variables, favoring grain yield (GY r^2^ = 0.73), pod weight (PW r^2^ = 0.77), seed viability (NVS r^2^ = 0.72), and efficiency in biomass partitioning (HI r^2^ = 0.57) (P < 0.05). Additionally, it contributed to reducing the formation of non-viable seeds (NNVS r^2^ = −0.59) (P < 0.05). However, during the mid-pod-filling stage (RSC_m_, Fig 7), where sugar concentrations in roots were lower, negative correlations (P < 0.05) were found with key variables such as grain yield (GY r^2^ = −0.57), pod weight (PW r^2^ = −0.54), and seed viability (NVS r^2^ = −0.58). This low sugar concentration in roots indicate the shift in assimilate movement toward pod and seed filling and influencing the formation of non-viable seeds (NNVS r^2^ = −0.70) (P < 0.05, Fig 7).

The leaf sugar concentration during flowering (LSC_f_, Fig 7) showed significant positive correlations (P < 0.05) with variables related to reproductive efficiency and yield, such as the number of viable seeds per plant (NVS, r² = 0.64), pod partitioning index (PPI, r² = 0.48), and pod harvest index (PHI, r² = 0.37). Positive correlations were also found with pod weight per plant (PW, r² = 0.69). In contrast, during mid-pod filling (LSC_m_, Fig 7), negative correlations were observed with pod stomatal density (PSD, r² = −0.32). The stem sugar concentration during flowering (SSC_f_, Fig 7) showed positive correlations (P < 0.05) with key agronomic variables such as grain yield (GY, r² = 0.68), canopy biomass (CB, r² = 0.78), pod weight per plant (PW, r² = 0.71), and harvest index (HI, r² = 0.52). It also showed positive correlations with reproductive variables like the number of viable seeds per plant (NVS, r² = 0.66) and viable seed weight per plant (VWS, r² = 0.70). However, during mid-pod filling stem sugar concentration (SSC_m_, Fig 7) showed negative correlations with variables associated with reproductive limitations, such as the number of non-viable seeds per pod (NNVSP, r² = −0.65). The pod sugar concentration during flowering (PSC_f_, Fig 7) exhibited strong positive correlations (P < 0.05) with variables directly linked to reproductive success, such as the number of viable seeds per pod (NVSP, r² = 0.53), pod weight per plant (PW, r² = 0.79), and grain yield (GY, r² = 0.76). Positive correlations were also observed with plant height (PH, r² = 0.27) and the number of pods per plant (NP, r² = 0.77). However, during mid-pod filling, pod sugar concentration (PSC_m_, Fig 7) showed negative correlations with the number of non-viable seeds per pod (NNVSP, r² = −0.75). Many variables related to chlorophyll content (RC, Fig 7), leaf temperature difference (LTD), chlorophyll fluorescence parameters (F_m_, F_0_, F_s_, F_v_/F_m_), and photosystem functioning parameters (ΦII, PSI_open_, Fig 7) were found to be positively correlated with variables related to grain yield and biomass partitioning. However, variables such as ΦNPQ and NPQ_t_, which are related to energy dissipation in the form of heat, were negatively correlated with most of the agronomic performance (yield and biomass partitioning) related variables. A similar situation occurred with the PSI_or_ and PSI_ox_ centers.

**Fig 8:**
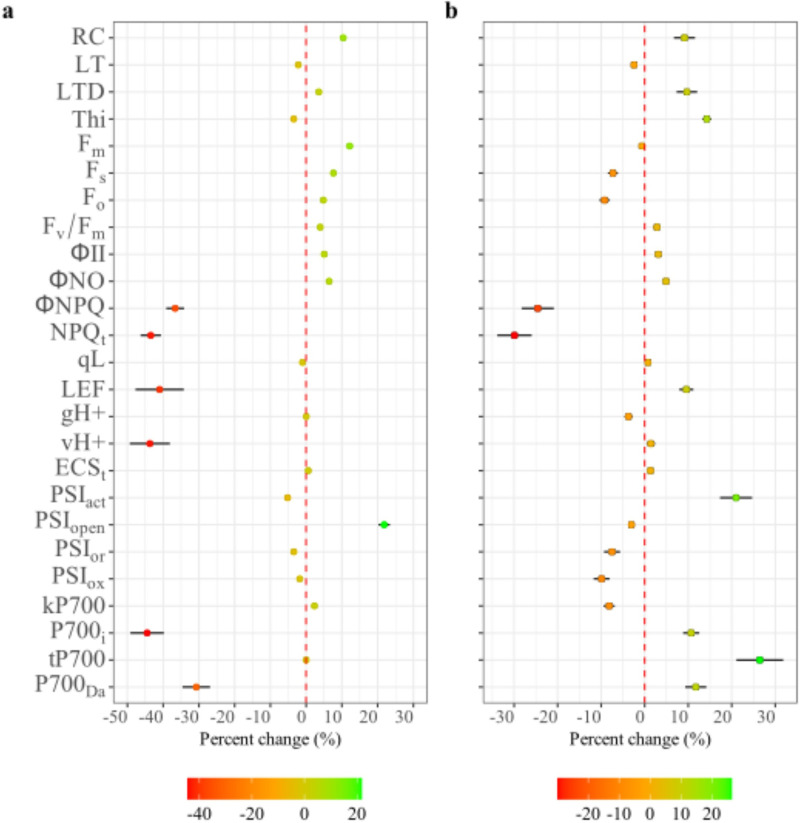
Percentage change by each factor of the different parameters of photosynthetic apparatus functioning in two common bean lines grown in acidic soil under high temperature conditions. a. Percentage difference between the phosphorus supply level P45 in relation to P0. b. Percentage difference between SEF 10 in relation to BFS 10. Red to green gradient means a positive and negative value between the analyzed items, the red dotted line denotes the zero value. RC: relative chlorophyll; LT: leaf temperature; LTD: leaf temperature difference; Thi: leaf thickness; F_m_: maximum fluorescence; F_0_: minimum fluorescence: F_v_: variable fluorescence; F_v_/F_m_, maximum quantum efficiency of PSII; ΦII: quantum yield of photosystem II; ΦNO: quantum yield of non-regulated; ΦNPQ: non-photochemical quenching; NPQ_t_: non-photochemical quenching total; qL: proportion of open PSII reaction centers; LEF: linear electron flow; gH + : proton conductivity of ATP synthases in the thylakoid membrane; vH + : the functioning of ATP synthase; ECS_t_: maximum amplitude of electrochromic shift; PSI_act_: total number of active PSI sites; PSI_open_: open state PSI sites; PSI_or_: reduced PSI fraction; PSI_ox_: fraction of oxidized PSI sites. _k_P700: electron transfer rate constant in P700; P700_i_: the initial electron transfer rate in steady-state P700; _t_P700: the electron transfer lifetime of P700; P700_Da_: the amplitude of P700 electron transfer during the light-dark transition.

On the other hand, when analyzing the changes between the factors (P0 vs. P45 and BFS 10 vs. SEF 10), significant changes were found in some variables (Fig 8). For instance, increasing the P supply (P45) significantly reduced the amount of energy dissipated as heat (ΦNPQ and NPQ_t_), as this energy was redirected to the operation of the photosynthetic apparatus (ΦII, Fig 8a). However, the increase in P supply also affected chlorophyll content and various variables related to the performance of the photosynthetic apparatus ((F_m_, F_0_, F_s_, F_v_/F_m_, Fig 8a). When comparing the differences between BFS 10 and SEF 10 (Fig 8b), it was found that the latter had a higher relative chlorophyll (RC) content as well as a greater capacity to dissipate excess heat (LTD, Fig 8b). In addition, SEF 10 had a higher proportion of active centers in PSI (PSI_act_), the initial electron transfer rate in steady-state P700 (P700_i_), the electron transfer lifetime of P700 (_t_P700), and the amplitude of P700 electron transfer during the light-dark transition (P700_Da_) compared to BFS 10 (Fig 8b).

### 3.6 Comparative differences in overall physiological performance between the two bean lines

The differences between the two bean lines (BFS 10, SEF 10) in their physiological response to increased P supply are shown in Table 1. Both lines showed physiological adaptations to cope with the combined low P and heat stress, but SEF 10 stands out for its higher leaf cooling capacity and photosynthetic efficiency under low P conditions, while BFS 10 exhibited higher sugar and P accumulation under high P supply conditions.

**Table 1:** Comparative differences in physiological response of two bean lines.

Characteristics	BFS 10	SEF 10
Leaf cooling capacity	Average leaf temperature difference (LTD) of 3.61°C	Average LTD of 3.98°C (better heat dissipation capacity)
Leaf temperature	Increase: 0.72°C (flowering) and 0.68°C (pod filling) higher than SEF 10	Decrease: 0.72°C (flowering) and 0.68°C (pod filling) lower than BFS 10
Photosynthetic efficiency (Fv/Fm)	Improves with high P (P45), reaching 0.72	Higher under low P (P0), reaching 0.67 compared to 0.59 in BFS 10
Energy allocation (ΦII)	Responds quickly to increased P, especially at P15 and P45	Maintains higher ΦII under heat stress and low P levels
Sugar accumulation	Higher accumulation in pods (60 mg g ¹) and stems (30 mg g ¹) with P45	Lower accumulation in pods (50 mg g ¹), but more efficient in chlorophyll
Leaf P concentration	Cumulative increase of 133% with high P (P45) treatment	Moderate increase of 77.8%; better efficiency with lower P under low P (P0) treatment
Heat dissipation mechanisms	Higher NPQ and NPQt during pod filling to protect from excess light	Better heat dissipation capacity, allowing higher photosynthetic efficiency
Genetic adaptation	Adapted to low-fertility soils with less tolerance to high temperature	Interspecific line derived from using *P. acutifolius*, with greater tolerance to high temperature

## 4. Discussion

It is evident that under the combined stress environment of acidic soil heat stress in which the two bean lines were grown, the increase in P supply to soil could have alleviated the effects of high temperature stress by improving leaf cooling capacity, the functioning of the photosynthetic apparatus, and the capacity to mobilize photoassimilates for pod and seed formation and seed filling. In our previous studies under the combined stress conditions of acidic soil and high temperature conditions [15,19,38] we observed a reduction in the growth rate and grain yield of both bean lines (BEF 10, SEF 10), specifically impacting the development of pods and seeds. In those studies, a mean night temperature higher than 25.9°C reduced the viability and formation of flower buds, as the number of flowers produced was affected by high temperature stress, which subsequently impacted the formation of pods and the mobilization of assimilates for seed filling. In general, the two bean lines are very good because they have the ability to adapt to the combined stress of acidic soils and high temperatures, but SEF 10 has an advantage over BFS 10, since it is the result of crosses using *Phaseolus acutifolius* which contributed genes for heat resistance [52] combined with the ability to adapt to the combined stresses of high temperatures and acidic soils [15,19,38]. In this study, we evaluated the influence of increasing P supply on different photosynthetic traits to analyze how photosynthetic adaptation at leaf level contributed to improved agronomic performance under combined acidic soil and high temperature stress.

### 4.1 Heat dissipation mechanisms in the canopy to cope with high temperatures under different levels of P supply to acidic soil

The environmental conditions during the study were characterized by average daytime and nighttime temperatures of 37°C and 29°C, respectively. These conditions significantly impacted the functioning of the photosynthetic apparatus, causing the evaluated two bean lines to adjust their leaf temperature (LT) within the canopy. Specifically, the LT values decreased by 3.6 to 4.0°C relative to the ambient temperature (T_a_). This temperature dissipation is linked to enhanced gas exchange (g_s_), which increases the rate of transpiration (E), leading to leaf cooling and improved carbon fixation at the cellular level [16,53]. The mechanism of temperature regulation through transpiration is crucial in common bean, as it helps maintain lower values of LT compared to the T_a_ [54]. In conditions of heat stress, the activation of heat shock proteins and changes in stomatal conductance are physiological adaptations that allow common bean to cope with high temperatures, reducing cellular damage and maintaining metabolic functions [55]. As a result, the plants were able to sustain photosynthetic activity and avoid damage from high temperatures, a phenomenon observed in both genotypes under the control treatment [53]. Recent studies have demonstrated that high-temperature stress can severely affect bean yield and quality, highlighting the need for effective nutrient management in stressed environments [56]. When P was supplied (in treatments P15, P30, P45), we found that increased P supply played a crucial role in supporting photosynthetic function. It is a key element in various photosynthetic processes, enhancing PSII performance by increasing the number of open reaction centers. This allows the genotypes to capture energy more efficiently, improve nutrient transport, reduce the impact of heat stress, and increase CO_2_ assimilation efficiency. Recent research indicates that effective P management improves yield and physiological performance in common beans under high temperatures [57]. Appiah-Kubi et al. [58] showed that P supplementation boosts heat stress resilience by enhancing antioxidant activities and minimizing oxidative damage. Additionally, Karavidas et al. [59] demonstrated that adequate P supply enhances photosynthetic performance and nutrient transport, alleviating heat stress in common beans. Consequently, this decreases latent heat transfer in the leaves during both phenological stages (flowering and mid-filling) [27].

The tolerance exhibited by the BFS 10 line under stress conditions is indicated by a more negative leaf temperature difference (LTD) in the upper leaves of the canopy, reflecting its ability to regulate leaf temperature despite direct exposure to air temperature and radiation, thereby maintaining photosynthetic function without restrictions [53]. In contrast, the SEF 10 line shows an increase in LTD (closer to zero) in its upper leaves while still maintaining physiological functions without adverse effects. The distinction between these lines may arise from the BFS 10 line’s adaptation to low fertility soils, whereas the SEF 10 line is derived from a cross of *Phaseolus vulgaris*, *P. acutifolius*, and *P. coccineus*, known for its high-temperature tolerance and adaptability to low P and high aluminum conditions [52]. Moreover, our findings indicate that in treatments with increased P supply, the LTD values increased by an average of 2°C across different canopy positions compared to the P0 treatment, suggesting that improved P allocation plays a crucial role in reducing heat transfer in the leaves of both genotypes. Notably, energy expenditure during the mid-pod filling stage exceeds that of the flowering stage, creating a microclimate within the canopy that sustains physiological processes and enhances photosynthate translocation for grain formation, thus increasing grain yield [15,19,38]. Enhanced leaf cooling is critical for heat tolerance in common bean, as demonstrated by Deva et al. [53], who found that leaf temperatures could be reduced by up to 5°C under heat stress, significantly improving photosynthetic performance by maintaining higher rates of carbon fixation. Suárez et al. [16] reinforced this by showing that leaf cooling allowed for a 20% increase in carbon assimilation efficiency, emphasizing that efficient temperature regulation is essential for optimizing photosynthetic processes.

Together, these studies underscore the significance of leaf cooling in enhancing heat tolerance and sustaining the photosynthetic apparatus under challenging environmental conditions. In addition, we found that in the treatments with increased P supply the LTD values increased on average 2ºC in the different positions of the canopy compared to the P0 treatment. This would indicate that the allocation of P influenced the decrease in heat transfer in the leaves of both genotypes. Likewise, the energy expenditure of the genotypes in the growth stage of mid-pod filling is greater than that presented at the beginning of the reproductive phase (flowering), a situation that generated conditions within the canopy to maintain physiological processes (better canopy microclimate) and hypothetically facilitating translocation of photosynthates for grain formation, thus increasing the grain yield [15,19,38]. Thus, we attribute that these phenotypic characteristics that both bean lines present, allow them to cope with high temperature stress conditions. However, we found that under increased P supply, they modify their adaptive responses in favor of facilitating their photosynthetic functioning, taking advantage of the favorable condition to counteract heat stress, for example, by increasing ATP production, which is necessary for CO_2_ fixation, thus improving their canopy development and grain production [60].

### 4.2 Improved phosphorus supply increases the energy dissipation mechanisms under high temperature stress

Under the heat stress conditions observed in the control treatment (P0), the maximum efficiency of PSII photochemistry (F_v_/F_m_) decreased, leading to an increase in heat dissipation mechanisms, specifically non-photochemical quenching (NPQ) and the total NPQ (NPQ_t_). This response was more pronounced during the mid-pod filling growth stage due to the higher energy expenditure associated with this phenological phase [61]. An increase in NPQ and NPQ_t_ is considered a protective mechanism against excess incident light, as it temporarily reduces the number of open reaction centers without modifying maximum photosynthetic efficiency [62]. Furthermore, chlorophyll content increased in the treatments with P supply; however, no significant variation was observed among the different P levels (P15, P30, P45) or by time of the day [63]. For instance, the BFS 10 line exhibited a pronounced decrease in chlorophyll content during the mid-pod filling growth stage, which may be related to P deficiency in the control treatment. This decrease could also be a strategy to prevent damage from excess light, as this genotype reduces chlorophyll content to protect PSII from potential radiation-induced harm [64,65].

The increase in P supply (P15, P30, P45) increased the efficiency of the photosynthetic machinery (F_v_/F_m_), due to a higher proportion of open PSII reaction centers, which contributed to ATP and NADPH production [27]. According to this, it was found that under heat stress conditions, as seen in the control treatment (P0), the LEF is inversely proportional to F_v_/F_m_ and ΦII, due to greater energy dissipation as heat (NPQ), which similarly affects LTD values. Under different P treatments, however, the LEF behavior changed, showing lower values. This is attributed to greater availability of ATP synthase in both lines [48], leading to changes in the thylakoid membrane as a photoprotective mechanism by dissipating excess excitation energy from the light-harvesting complexes to avoid PSII over-excitation [66]. Furthermore, we suggest that increase in P supply may help create an ATP and NADPH sink, potentially reducing the need for thermal energy dissipation and improving the response to heat stress [67].

Some key regulatory mechanisms involve the proton gradient across the thylakoid membrane generated by light-driven electron transfer reactions through linear electron flow (LEF) and cyclic electron flow (CEF) [68]. Specifically, LEF is coupled to the generation of a proton gradient across the thylakoid membrane (ΔpH), driving ATP synthesis to fuel primary metabolism, including the Calvin cycle and photorespiration [69]. Under stress conditions, the ΔpH acts as a key regulator of photosynthesis, activating non-photochemical quenching (NPQ) to dissipate excess light energy [70], reducing the excitation energy pressure on PSI and PSII reaction centers [35]. The data from this study showed that under both phenological stages evaluated (flowering and mid-pod filling), both genotypes exhibited similar qL behavior across all treatments, indicating that PSII did not experience limitations or electron accumulation in Q_A_. This implies that electron transfer was not compromised on the PSI acceptor side, and most reaction centers were open[48]. Moreover, BFS 10 and SEF 10 increased their ΦII, indicating activation of non-regulated non-photochemical losses (ΦNO), which could be related to the small changes observed in LTD, as a protective mechanism in response to high temperature, preserving the functionality of the photosynthetic apparatus [71]. In this way, we found that the two lines, BFS 10 and SEF 10, used different balancing and adjustment mechanisms in the performance of PSI and PSII as a means of protecting the photosynthetic apparatus under heat stress [72].

### 4.3 Increase in P supply facilitates photosynthetic performance and improves photosynthate mobilization to pods under high temperature stress

A key aspect of improving total P concentration in leaves under high temperature stress conditions is its ability to increase the mobilization of photoassimilates, particularly sugars, to the reproductive organs. This process is crucial for improving heat tolerance, as it ensures the supply of energy and carbon for seed development. In common bean, this redistribution of photoassimilates is evident during mid-pod filling, especially under drought in fertile soil with adequate P supply [9,73]. In the present study, P45 treatment increased sugar concentration in pods up to 60 mg g^-1^ compared to 20 mg g^-1^ under P deficient conditions (P0), highlighting the importance of this nutrient in improving partitioning of photoassimilates. Studies such as those by Fageria et al. [40] showed that adequate P supply significantly increases carbohydrate accumulation in pods, with a 30% increase in pod number per plant and a 25% increase in seed weight under high temperature stress conditions. This is attributed to a greater efficiency in the partitioning of photoassimilates to the reproductive organs, which improves yield stability. Likewise, Hungria and Vargas [74] reported that increased P supply improves nitrogen fixation and photosynthetic efficiency in common bean, which allows greater biomass accumulation and better mobilization of photosynthates to the pods. Under heat stress conditions, these improvements translate into yield increases of up to 20% compared to P-deficient plants. Beebe et al. [1] noted that tolerance to low P supply contributes to yield stabilization in common bean grown in warm regions by improving the mobility of photoassimilates to pods and seeds.

The P also acts as a metabolic regulator that improves resource use efficiency and carbon partitioning, even under different abiotic stress conditions [22,75]. In common bean, increase in P supply not only increases photoassimilates production, but also allows its accumulation in reproductive organs as a metabolic buffering strategy, reducing the impact of heat stress [76]. This mechanism is particularly important during pod filling, a stage when photoassimilates demand is critical to ensure the development of viable seeds [28]. This effect is observed in other crops such as maize (*Zea mays*), where increased P supply improves nutrient acquisition and stress tolerance [77]. In rice (*Oryza sativa*), Sharma et al. [32] reported that P application improves carbohydrate accumulation in grains during critical stages such as flowering and grain filling, resulting in increased tolerance to heat stress. Additionally, Ghosh et al. [78] found that increased P supply increases the mobility of photoassimilates and reduces yield losses in rice grown under high temperatures. In soybean (*Glycine max*), Khan et al. [20]showed that increase in P supply improves photosynthetic efficiency and carbohydrate partitioning to pods, resulting in higher number of grains per pod under heat stress conditions. In tobacco (*Nicotiana tabacum*), Xu et al. [79] showed that increased P supply improves heat tolerance by stimulating the activity of enzymes such as invertase, which facilitates the partitioning of sugars to the ears. This resulted in higher kernel weight and a smaller drop in yield under heat stress. Similar results were reported by Wahid et al. [28], who found that increase in P supply increases carbohydrate accumulation and photosynthetic efficiency, which mitigates the negative effects of heat on grain filling. The above studies clearly indicate that increased P supply plays a crucial role in mitigating high temperature stress by increasing the mobility of photoassimilates to the reproductive organs. This results in greater yield stability and better adaptation to heat stress conditions.

On the other hand, differences among bean lines influenced the ability of plants to tolerate heat stress. For example, some lines have shown a higher efficiency in P utilization, which allows them to maintain a higher accumulation of photosynthates and a better translocation of photosynthates to pods, even under high temperature conditions [7,80]. In this sense, BFS 10 reached to sugar levels of 60 mg g^-1^ in pods with P45, while SEF 10 only reached 50 mg g^-1^. These differences could be associated with genetic variation in P uptake and utilization capacity, as well as in photosynthate transport efficiency. Results from this study agree with the published reports on the role of improved P supply on mitigating the heat stress effects on grain yield. Thus, it is possible that proper management of fertilization schemes in crops can help to cope with high temperature stress conditions [75].

The results on total leaf P concentration under different levels of P supply reflect contrasting strategies between the two genotypes (BFS 10 and SEF 10), consistent with previous studies on common bean responses to P supply, particularly in acidic and nutrient-poor soils. The increase in leaf P concentration in both genotypes, from P0 to P45 treatments, shows a typical dose-response relationship. The BFS 10 exhibited an accumulated increase of 133%, indicating a greater capacity for P accumulation under high P availability, likely due to greater root expansion or efficient regulation of P transporters [81]. On the other hand, SEF 10 showed a more moderate increase (77.8%), which could be related to a conservative physiological strategy, optimizing the use of absorbed P rather than accumulating it in large amounts. This behavior aligns with the findings of Ramaekers et al. [82], who reported that P-efficient bean genotypes tend to maintain relatively constant concentrations of this nutrient, even under high availability, prioritizing its internal redistribution to key organs such as young leaves and reproductive tissues. Under low P supply conditions (P0 and PSOM), SEF 10 outperformed BFS 10 in leaf P, with differences of 20% and 15%, respectively. This result suggests that SEF 10 possesses more efficient mechanisms for P acquisition or utilization under P limiting conditions. These mechanisms may include adaptations such as organic acid exudation, association with mycorrhizae, and aluminum tolerance, which have been widely documented by Rao et al. [83]. Such adaptations are key characteristics of P-efficient genotypes, as documented in bean varieties adapted to acidic and P-deficient soils in Latin America and Africa [6]. At higher P supply levels (P30 and P45), the differences between the two bean lines were minimal. This result indicates that under P-sufficient conditions, both bean lines are capable of absorbing and accumulating similar amounts of this nutrient. However, the fact that BFS 10 experiences a more pronounced increase in leaf P concentration suggests that this line has a greater storage capacity, possibly as a strategy to ensure internal P availability during critical developmental stages, such as flowering and mid-pod filling [39].

### 4.4 Increase in phosphorus supply improves photosynthetic and agronomic performance under high temperature stress

Ambient temperature and nutrient availability, such P, are among the main drivers of plant productivity, and changes in these factors significantly influence plant adaptation in each environment [65]. When analyzing the correlations between the evaluated variables, we found that the energy utilization mechanisms in both bean lines are directed toward maintaining and enhancing photochemical pathways under stress conditions. For both BFS 10 and SEF 10, a positive correlation was found between LTD and GY as well as with different agronomic and biomass partitioning variables. These results are contrary to those reported by Deva et al. [53] and Suárez et al. [16] who have reported that under heat stress conditions the leaf temperature difference (LTD) becomes more negative due to its cooling capacity which correlates negatively with GY. This behavior in relation to the positive correlation between LTD and GY is due to enough P supply that influences the optimal functioning of the photosynthetic apparatus even under high temperature conditions. That is, the addition of P allows them to increase ATP and NADPH production, regulating the mechanism of the thylakoid membrane and maintaining electron transfer through the LEF. Additionally, both bean lines dedicated a significantly larger proportion of energy to other non-photochemical dissipation processes (ΦNO) and a smaller proportion to ΦNPQ, translating to greater photoprotective capacity while maintaining CO_2_ fixation [48,84].

The leaf P concentration (LPC) showed positive correlations with agronomic variables such as grain yield (GY), pod weight (PW), and the number of viable seeds (NVS), as well as with different dry matter partitioning indices (PPI, PHI, HI). This suggests that higher P concentration in leaves is associated with an increased ability of plants to sustain essential metabolic processes, such as photosynthesis, and to allocate resources towards the production of reproductive organs [10,22,31,40,58]. For instance, the positive correlation between LPC and PW indicates that increased P supply increases grain production through enhanced allocation biomass to pods, reflecting greater efficiency in resource allocation towards reproductive organs [4,21,77]. Additionally, LPC exhibited a positive correlation with NVS, implying that higher leaf P concentration promotes the formation of viable seeds, possibly due to its role in ATP synthesis and the regulation of metabolic processes during seed formation and filling [11,60]. This suggests that supply of adequate P to soil can reduce the formation of non-viable seeds, thereby improving both the quality and quantity of production [38]. On the other hand, leaf P concentration showed negative correlations with variables related to number of non-viable seeds and number of non-viable seeds per pod (NNVS, NNVSP), indicating that insufficient supply of P from soil may increase the formation of non-viable seeds. This P supply effect could be attributed to better energy and nutrient availability during reproductive development, ultimately enhancing production quality and quantity.

The above physiological responses resulting from increased P supply under the combined acidic soil and high temperature stress conditions significantly influenced the mobilization of photoassimilates from canopy biomass (CB) to pod (PNA) and seed (SNA) production as reflected through different dry matter partitioning indices (PPI, HI and PHI) [15,19,38]. The results of this study indicate that selection based on physiological traits, where the supply of P to acidic soil plays an important role in plant growth, development, and yield stability in the face of climate change, could contribute to improved yield gains in bean breeding programs [85]. Furthermore, the two common bean lines (SEF 10, BEF 10) that are photosynthetically adapted to combined stress conditions of acidic soil and high temperature could also serve as a valuable genetic resource for improving grain yield in the face of climate change. It has also been reported that increase in P supply to acidic soil has a positive impact on the functioning of the photosynthetic apparatus in other types of abiotic stress. For example, P applied as foliar fertilization before water stress has a positive impact on the recovery of photosynthesis and yield, especially in common bean genotype A320 [86]. This effect is due to the improvement in P metabolism, ATP regeneration, and reduction of NPQ mechanisms during recovery, which facilitate the maintenance of photosynthetic efficiency under stress conditions. Likewise, previous research showed genotypic differences in common bean: two improved lines, NCB 226 and SEF 60 exhibited greater photosynthetic resilience under low P stress by maintaining better values of Vc_max_, J and TPU compared to commercial genotypes such as DOR 390 [87].

This study provides several relevant novelties on how increase in P supply can mitigate the effects of the combined stress of high temperatures and acid soils. First, significant improvements in leaf cooling capacity (LTD) were evidenced, which contributed to more efficient thermal regulation. This effect was most evident during the mid-pod filling stage, when plant energy demand is the highest. In addition, improved functioning of the photosynthetic apparatus was observed, particularly in PSII efficiency (F_v_/F_m_) and linear electron flow (LEF). These improvements were accompanied by increased mobilization of photoassimilates to reproductive organs, such as pods and seeds, resulting in increased yield. An important novelty lies in how P supply influenced energy redistribution within the photosynthetic apparatus. A decrease in energy dissipation as heat (NPQ and NPQ_t_) was observed with increasing P supply, redirecting this energy toward photosynthetic processes (ΦII). This energy adjustment allowed improving photosynthetic efficiency even under high temperatures, a mechanism that had not been previously documented so clearly in beans subjected to combined stress.

This study also provides novel data on how P supply enhances the translocation of sugars from the canopy to reproductive organs. For example, an increase of up to 200% in sugar concentration in pods was reported under P45 treatment compared to P0. At the genotypic level, BFS 10 showed a higher level of sugars in pods (60 mg g^-1^) than SEF 10 (50 mg g^-1^), highlighting differences in photoassimilate transport efficiency among advanced bean lines. Pod partitioning index (PPI) and harvest index (HI) improved with increasing P, indicating that increased P supply facilitates resource mobilization to reproductive organs, improving grain yield [38]. In addition, this work documents how P supply facilitates the regeneration of ATP and NADPH, essential for maintaining photosynthesis under heat stress. This included an increase in the proportion of open reaction centers (qL), indicating efficient electron transfer and reduced accumulation in Q_A_, features that contribute to increased photosynthetic resilience.

The results from this study have direct implications for breeding programs and agronomic management strategies. The SEF 10 and BFS 10 lines stand out as valuable genetic resources to cope with climate change, combining tolerance to heat and acid soils. For example, SEF 10 showed higher leaf cooling capacity and photosynthetic efficiency under conditions of low P availability, which is attributed to its genetic background of *P. acutifolius*, which is known for its higher level of tolerance to heat. On the other hand, BFS 10 exhibited higher P accumulation in leaves under high P supply levels, suggesting an internal P storage strategy that ensures availability during critical growth stages, such as pod filling. Results from this study together with the results from previous study [38] demonstrate how increase in P supply can modify photosynthetic responses of two bean lines and improve their agronomic performance under combined stress conditions of high temperature and acidic soils. In addition, this study also highlights differences between two adapted common bean lines in leaf cooling mechanisms, photosynthetic efficiency, and photoassimilates mobilization, providing a valuable framework for improving bean productivity under climatic and edaphic stress conditions.

## 5. Conclusion

In this study, the average daytime and nighttime temperatures were 37°C and 29°C, respectively, a condition that significantly affected the functioning of the photosynthetic apparatus in the evaluated two bean lines (SEF 10, BEF 10) under increased P supply. As an adaptation mechanism to high temperatures under increased P supply, the canopy temperature was adjusted, reducing leaf temperature by 3.6 to 4.0°C compared to the ambient temperature. This allowed the leaves to cool, directing a greater fraction of energy to the photosynthetic machinery. Increased P supply under these high-temperature conditions also increased ATP production, improved PSII photochemical efficiency (F_v_/F_m_), and reduced the need for heat dissipation. It was found that SEF 10 under high temperature conditions can dissipate excess energy in the form of heat compared to BFS 10, a trait that allows it to have a higher photosynthetic efficiency by having a better ability to cool the leaves. This leaf cooling ability allowed for a higher value of photosynthetic efficiency (F_v_/F_m_) as well as better performance in energy allocation to photosynthesis. Increasing P supply increased leaf P concentration and improved the photosynthetic efficiency and sugar accumulation in pods and seeds facilitating improved seed yield in both common bean lines. Thus, increasing the supply of P to acidic soil increased photosynthetic adaptation and agronomic performance of both common bean lines (SEF 10 and BFS 10) under high temperature stress. Both bean lines contain desirable traits needed to be considered by the bean breeding programs for further improvement in physiological performance under combined stress conditions of acid soil and high temperature stress. We suggest that between the two lines, BSF 10 is better suited if P supply is insufficient in acid soil, and SEF 10 is better if P supply is sufficient but temperature is high.

## Supporting information

S1 Table Physiological variables, sugar and phosphorus content in advanced common bean lines. (XLSX)
